# Environmental Factors Affecting Monoterpene Emissions from Terrestrial Vegetation

**DOI:** 10.3390/plants12173146

**Published:** 2023-08-31

**Authors:** Tanzil Gaffar Malik, Lokesh Kumar Sahu, Mansi Gupta, Bilal Ahmad Mir, Triratnesh Gajbhiye, Rashmi Dubey, Andrea Clavijo McCormick, Sudhir Kumar Pandey

**Affiliations:** 1 Department of Botany, Guru Ghasidas Central University, Bilaspur 495009, Chhattisgarh, India; tanzil@prl.res.in; 2 Space and Atmospheric Sciences Division, Physical Research Laboratory, Ahmedabad 380009, Gujarat, India; mansigupta@prl.res.in; 3 Department of Botany, University of Kashmir (North Campus), Delina, Baramulla 193103, Jammu & Kashmir, India; meerbilal82@gmail.com; 4 Department of Botany, Govt. Shankar Sao Patel College Waraseoni, Waraseoni 481331, Madhya Pradesh, India; triratnesh@gmail.com; 5 Department of Chemistry, L.B.S. College, Baloda 495559, Chhattisgarh, India; rashmibhu@gmail.com; 6 School of Agriculture and Environment, Massey University, Palmerston North 4472, New Zealand

**Keywords:** monoterpenoid, abiotic factors, season, temperature, light, pinene

## Abstract

Monoterpenes are volatile organic compounds that play important roles in atmospheric chemistry, plant physiology, communication, and defense. This review compiles the monoterpene emission flux data reported for different regions and plant species and highlights the role of abiotic environmental factors in controlling the emissions of biogenic monoterpenes and their emission fluxes for terrestrial plant species (including seasonal variations). Previous studies have demonstrated the role and importance of ambient air temperature and light in controlling monoterpene emissions, likely contributing to higher monoterpene emissions during the summer season in temperate regions. In addition to light and temperature dependence, other important environmental variables such as carbon dioxide (CO_2_), ozone (O_3_), soil moisture, and nutrient availability are also known to influence monoterpene emissions rates, but the information available is still limited. Throughout the paper, we identify knowledge gaps and provide recommendations for future studies.

## 1. Introduction

All terrestrial plants emit biogenic volatile organic compounds (BVOCs), relatively small chain hydrocarbon compounds that have lower boiling points and evaporate easily. These compounds play an important role in atmospheric chemistry [1], plant physiology [2], plant defense and competition [3,4,5,6], and communication between plants and other organisms [7,8,9].

Monoterpenes are the second most dominant group of BVOCs after isoprene, with an estimated global annual emission rate of 107.5 Tg C yr^−1^ contributing ~12% to the global BVOC budget [10]. Monoterpenes are generally derived from the condensation of two isoprene (C_5_H_8_) units and include a variety of well-known compounds including pinene, linalool, and limonene among others [11]. Biosynthesis of monoterpenes is catalyzed by monoterpene synthases (cyclases), which convert the universal precursor geranyl diphosphate (GDP) to the parent structures of the various monoterpene groups. De novo synthesis is light-dependent and can occur in the cytosol through the mevalonic pathway (MEV) or in the chloroplast, through the methylerythritol phosphate (MEP) pathway [11,12].

Different plant species will have different enzymes leading to the formation of specific monoterpenes (monoterpene synthases), leading to a huge diversity of these plant natural products across the plant kingdom [11,12]. For instance, *α*-pinene and *β*-pinene make up most monoterpene emissions from oaks and conifers [13,14,15,16], while *E*-β-ocimene is commonly released by plants of the *Salicaceae* family [17,18,19]. Although a wide spectrum of monoterpenes is emitted by tree species [2,20], considerable emissions of some compounds (e.g., *α*-pinene and *β*-pinene, Δ^3^-carene, limonene, etc.) are also reported from *Poaceae* species as well as from rice, maize, bamboo, and other grasses [21,22,23,24,25,26]. Besides the emission of monoterpenes from the tree and grass species, over the last two decades there has been increasing work investigating monoterpene emissions in other crops [27,28], and horticultural species [29,30,31,32,33,34], as well as ornamental plants and invasive alien species [27,28,35,36].

The emission of BVOCs varies in time and intensity in response to abiotic factors such as temperature, light intensity, CO_2_, O_3_,and O_2_ concentrations, but the exact mechanisms behind these responses are not yet fully understood [19,37]. However, monoterpene emissions have been reported to have distinct dependencies on light and temperature compared to those reported for other BVOCs. This is attributed to the ability of some plants to store them and their high solubility in water (such as linalool) [38,39,40]. Terpenes can be produced de novo and released immediately or stored in non-specific internal pools or specialized endogenous and exogenous structures such as resin secretory structures and glandular trichomes [41,42,43].

The emissions of stored monoterpenes are mainly temperature dependent, while the non-stored monoterpenes are believed to be dependent on both temperature and light [41]. Moreover, the stored monoterpene emissions are also influenced by other factors such as humidity, diffusion resistance, cell wall, membrane thickness, and pool storage size [43,44,45,46]. In the next sections, we provide an overview of the abiotic environmental factors affecting monoterpene emissions, including putative mechanisms, and identify knowledge gaps to be addressed by future research.

## 2. Environmental Factors Controlling Biogenic Monoterpene Emission

**Figure 1:**
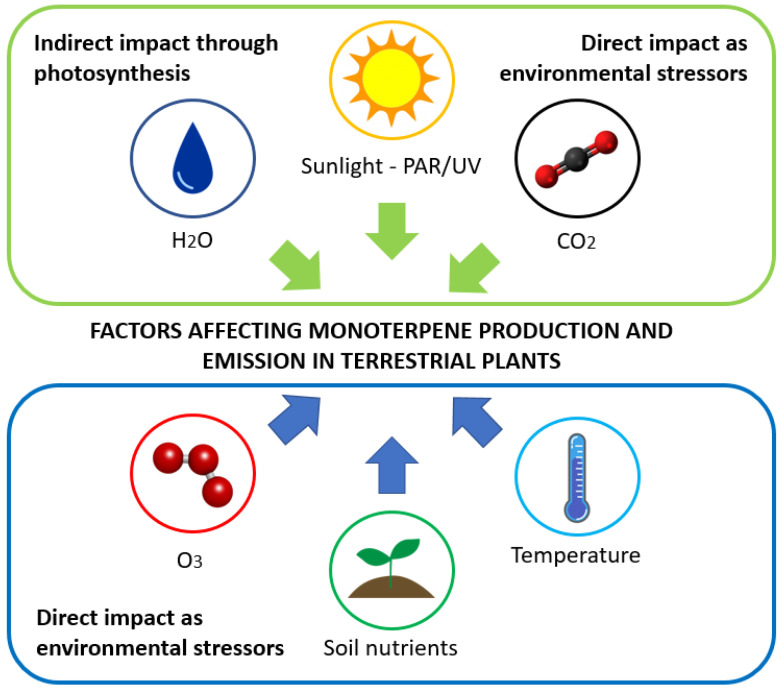
Schematic diagram of the factors affecting monoterpene production and emission from terrestrial plants.

Abiotic drivers, such as temperature, light, humidity, CO_2_ concentration, soil nutrients, etc., have been reported to influence monoterpene emissions from various plant species [27,47,48,49,50,51]. A summary of these factors and their impact on monoterpene production and emission are shown in Figure 1.

### 2.1. Temperature

Temperature is one of the most important abiotic drivers controlling monoterpene emission from plants [52,53,54,55]. It is well established that the variation in ambient temperature leads to the variations in monoterpene emissions from different plants [56]. Pioneer studies in coniferous plants (in boreal forests), already described monoterpene storage and noted that emissions increased with the rise in ambient temperature [57,58,59]. More recently, other investigators have confirmed those results, i.e., the response of monoterpene emission is positively correlated with temperature in coniferous plants [60,61,62]. However, the investigators have been unable to fully grasp the mechanism responsible for the release from plant storage. Therefore, further research in this area is needed to entirely understand the mechanisms behind monoterpene emissions.

On the other hand, de novo synthesized monoterpene emissions have been better characterized or parameterized. The de novo-based mechanism shows the highest emissions at the optimum temperature ranges of 37–40 °C [63]. For instance, Song et al. [64] have described the de novo monoterpene emission from *Quercus ilex* L. as a function of temperature. The highest emission rate of monoterpenes (5–25 µg g^−1^ h^−1^) were measured at ~40 °C and then gradual declines as temperature rises above 45 °C were noted. The decrease at very high temperatures could be due to enzyme (monoterpene synthase) inactivation during biosynthesis, raising their vapor pressure and decreasing the resistance of emission pathways [65,66,67]. Typically, the emission rate of monoterpenes is determined to increase exponentially with increasing temperature. This can be explained by the monoterpene storage pool linking the emissions to monoterpene volatility and Henry’s law constant [55].

Monoterpene emission can also be influenced by thermal stress/heat stress (when plants are exposed to a high temperature that affects some physiological processes) [68]. Due to heat stress, stomata open and monoterpenes are likely to be released into the atmosphere immediately after their synthesis from non-storage tissues [69]. Brilli et al. [70] suggested that heat stress induces monoterpene emissions rather than isoprene. Whereas, significant increase in monoterpene emissions have been reported in storage pools of conifers such as Scots pine and Norway spruce [71], tomato (*Solanum lycopersicum* L.) [72,73,74]. The non-storage pools for monoterpenes from Mediterranean species like European beech and Palestine oak (*Quercus calliprinos* L.) showed a decline of de novo monoterpene emission under heat stress.

Apart from heat stress, the monoterpene emissions are also altered during and after cold stress. The cold stress effect was found to be antagonistic to the heat stress for monoterpene emission in the case of *Solanum lycopersicum* plants [74]. Overall, the studies clearly indicate that the monoterpene emissions under thermal stress conditions depend on the plant species and its ability to store monoterpenes, but also on the experimental setup [56].

### 2.2. Sunlight

Sunlight has been reported to be another important factor, which can govern the BVOC emission pattern from different plant species [67,75,76,77,78]. The light-dependent emissions of BVOCs from light-grown plants show a particularly strong response compared to the shade-grown plants [53,79,80,81].

At present, there are only a few studies, which have carried out the experiments demonstrating the effect of light intensity on monoterpene emissions for some specific plant species [2,57,82,83]. However, Tingey et al. [65] had reported the influence of the light in the monoterpene emissions based on field experiments under ambient light regimes, but a direct dependence on light was not conclusively established. Temperature and radiation are the most important drivers for photosynthesis and thus for the provision of energy as well as BVOC precursor compounds [84]. Thus, all de novo emissions somehow depend on these two influences in combination. We distinguish these from emissions linked to the release from specific storage structures mainly by passive diffusion, which have a direct dependency on temperature but not on light [65].

The above canopy and branch level measurement-based studies reveal that the non-storage (de novo) monoterpene emissions are strongly light dependent [16,50,60,83,85,86,87,88]. For example, for a set value of photosynthetic photon flux density (PPDF) at 1000 μmol m^−2^ s^−1^, the monoterpene emission from *Quercus phillyreoides* A. Gray increased slowly and reached a constant after ~2 h. However, for lower values of PPDF and a constant temperature of 25 °C, the monoterpene emission rate started to decrease at a steady state within 20 min [86].

The evergreen oak *Q. coccifera* L. widespread in Mediterranean shrublands, was found to show light-dependent emissions of more than 50 BVOC species except for green leaf volatiles (GLVs) [71]. Among them, about 90% were non-oxygenated monoterpenes, and the rest were oxygenated monoterpenes and sesquiterpenes. The investigators have constructed the light-dependent curves for different isoprenoids, i.e., non-oxygenated monoterpenes, oxygenated monoterpenes, and sesquiterpenes. At a constant temperature of 30 °C, emissions of isoprenoids increased as PPFDs increased from 600 to 1500 μmol m^−2^ s^−1^. However, non-oxygenated monoterpene emission rates decreased at the higher PPFD values beyond 1500 μmol m^−2^ s^−1^ (up to saturation level). In this study, isoprenoid emissions were not or hardly detected at night confirming the light-dependent emissions from *Q. coccifera* L.

Researchers were able to derive the light-dependent photosynthesis and emission rate curves of monoterpenes for *Cecropia sciadophylla* (a common pioneer tree species in the Amazon Basin) at a constant leaf temperature of 30 °C [88]. A maximum total monoterpene emission rate of 35.8 nmol m^−2^ s^−1^ was observed at a maximum photosynthetically active radiation (PAR) of 2000 μmol m^−2^ s^−1^ and a leaf temperature of 30 °C. Among monoterpenes, trans-*β*-ocimene had a maximum emission rate of 24.5 nmol m^−2^ s^−1^ and a maximum photosynthesis rate of 19.0 μmol m^−2^ s^−1^. The other light-dependent monoterpenes include cis-*β*-ocimene (6.8 nmol m^−2^ s^−1^), *α*-pinene (1.5 nmol m^−2^ s^−1^), *β*-pinene (0.5 nmol m^−2^ s^−1^), *β*-myrcene (0.23 nmol m^−2^ s^−1^), and sabinene (0.16 nmol m^−2^ s^−1^).

In three dominant coniferous tree species (*Cryptomeria japonica* (Thunb. ex L.f.) D.Don, *Chamaecyparis obtusa* (Siebold and Zucc.) Endl. and *Pinus densiflora* (Siebold and Zucc.)) found in Japan, Nishimura et al. [83] have reported the strong light-dependent emissions of dominant monoterpene species. For *C. japonica*, the emission rates of *α*-Pinene, *β*-Pinene, and *α*-Phellandrene accounted for 61%; for *C. obtusa*, *α*-Pinene, *β*-Pinene, and D-Limonene accounted for 63%; and for *P. densiflora*, *α*-Pinene, *β*-Pinene, and *β*-Myrcene accounted for 95% of the total monoterpene. Similarly, emissions of *α*-pinene, sabinene, *β*-pinene, myrcene, and limonene from *Q. ilex* L. are strongly affected by light intensity and leaf temperature [75,79,89,90,91]. Despite knowledge about the light intensity-monoterpene correlation, the effect of light on the emissions from non-woody plant species needs further investigation.

Besides PAR, the sun also emits ultraviolet 100–400 nm (UV) and infrared (IR) radiation 780 nm and 1 mm, which may also influence monoterpene emissions. And there is an increasing body of evidence suggesting that UV radiation can significantly impact monoterpene emissions [92,93,94]. The effect of IR has not been extensively explored and requires further investigation.

### 2.3. Other Factors

In addition to temperature and light, other factors such as soil moisture, nutrients, humidity, O_3_, and CO_2_ concentrations can affect the monoterpene emissions, but studies on these aspects are scarce [95,96,97,98,99,100,101].

Soil moisture alters the de novo emission of monoterpene from some plants (European beech, Holm oak, Scots pine, and Norway spruce), whose emission is also known to be highly dependent on temperature and light intensity (PAR) [46]. The volumetric water content of the soil has been used as a reference quantity to parameterize the dependence of monoterpene emissions on soil moisture and to characterize the severity of the drought. It has been found that monoterpene emissions increase during mild drought and decrease during severe drought [46]. Mu et al. [98] studied the effect of soil moisture (i.e., drought) on isoprenoid emission from the two dominant Mediterranean species: *Erica multiflora* L. in a Garraf shrubland and *Q. ilex* L. in a Prades forest in Catalonia (Spain). Drought and control plots were classified on the basis of the covering and non-covering of transparent and waterproof plastic curtains over the plants and soil during rain for four seasons. When they were compared with controls, the drought conditions decreased soil moisture by ranging between 1.3% in the winter mornings and 14.7% at midday in *E. multiflora* L. (in a Garraf shrubland). Similarly, in *Q. ilex* L. (in a Prades forest), the soil moisture decreases between 21.0% in the winter mornings and 48.8% at midday. Isoprene, limonene, and *α*-pinene were the most dominant terpenes found in *E. multiflora* L. (limonene and *α*-pinene accounted for a significant portion of 80–84% of total emissions), while isoprene was not recorded in the case of *Q. ilex* L. Compound *α*-pinene increased 39.7% in winter mornings and 68.0% limonene increased at midday during drought treatments when compared to control treatments in *Q. ilex* L. A similar pattern was observed in the case of *E. multiflora* L., though having different emission values. The authors noted that the differences in emission rates between control treatments and drought conditions could be due to soil moisture variability.

The overall evidence is non-conclusive, with some studies reporting that drought has a positive effect on plant monoterpenoid emissions [102,103,104], but some reports contradict this (i.e., monoterpene emissions decrease dramatically under high drought conditions) [105,106,107]. It is conceivable that the intensity and duration of drought will have different impacts, as well as the drought-tolerance of the plant species. Therefore, more research is needed to explore this in depth.

The ambient air O_3_ concentration is a critical factor for controlling the VOC emissions from the plants. However, little is known about the impact of elevated ozone concentrations (long-term exposure) on the release of BVOCs [108]. Several studies report that the BVOC emissions from ozone-stressed plants are orders of magnitude higher than those from non-stressed plants [98,109,110,111]. However, studies exist showing no effect, e.g., Mochizuki et al. [112] reported that elevated O_3_ had no effect on the monoterpene of the hybrid larch F1 (*Larix gmelinii* var. *japonica* L. *kaempferi*).

Recently, Miyama et al. [108] reported that the monoterpene emission rate of ozone-exposed plants (*C. japonica*) was higher than that of non-exposed plants. They found that the basal emission rate of three clones of *C. japonica* (*C. japonica* ‘Donden’, *C. japonica* ‘Kawazu’, and *C. japonica* ‘Yakushima’) increased with long-exposure of O_3_. In the cases of ozone exposed cultivars ‘Donden’ and ‘Yakushima’, the composition of monoterpene compounds did not show significant differences with the non-exposed ones. However, the composition ratio of sabinene was increased from 25% to 75% in cultivar ‘Kawazu’, among others [108]. According to Mochizuki et al. [109], ozone-exposed plants stimulate monoterpene emission more than non-ozone-exposed plants. Hence, these studies revealed that the emissions of monoterpene with exposure to O_3_ are likely species-specific.

The effects of ambient CO_2_ concentration on monoterpene emissions of some dominant Indian tropical plants were examined by Malik et al. [39]. The results suggested no statistically significant effect on monoterpene emissions. However, one of the common species, i.e., *Eucalyptus globulus* Labill., showed a significant positive correlation (r = 0.69) with the ambient CO_2_ concentration in the summer season [39]. In another study, monoterpene emissions from *Cryptomeria japonica* clone saplings grown under control, ambient, elevated CO_2_, and at varying soil water content (SWC) concentrations were measured [96]. The results indicated that elevated concentrations of CO_2_ under control significantly affect the emissions of monoterpene from *Cryptomeria japonica*.

In contrast, a negative correlation between the monoterpene emissions and CO_2_ concentration has been reported for several plants [110,111,112]. When monoterpene emissions from *Q. ilex* species were measured under elevated CO_2_ concentrations [106], the emissions of the three most abundantly emitted monoterpenes (*α*-pinene, sabinene, and *β*-pinene) were inhibited by approximately 68%. However, the emission of minor compounds, i.e., limonene, was found to be increased at elevated CO_2_. Therefore, it is necessary to evaluate the dependence of different monoterpenes under varying CO_2_ concentrations.

The role of nutrient availability in regulating monoterpene emissions is less known. However, Fernández-Martnez [113] studied the isoprenoids (both isoprene and monoterpene) emissions in response to foliar nitrogen (N) and phosphorus (P) concentrations for 113 plant species and found differences in monoterpene emissions in association with different nutrients. This is an interesting finding, suggesting the possibility that N and P might be good predictors for inducing isoprenoid emissions. Thus, further studies are required to elucidate the role of other individual nutrients on monoterpene emissions.

We acknowledge that other factors, such as water vapor concentration (humidity) and aerosol compounds, could also influence the monoterpene emission patterns, but there is limited information available and therefore these are not considered in this review.

## 3. Seasonal Influences and Mechanisms Underlying Emission Patterns

In recent decades, many efforts have been made to explore the seasonality in emission rates of monoterpenes from different plant species in temperate latitudes [47,114,115,116,117,118,119]. This includes the complexity of interacting environmental factors. Previous studies in different plant species or even the same plant species in different regions revealed the significant seasonal variations of monoterpene emission [47,116,120,121,122,123,124]

As an example, we provide a summary (non-exhaustive) of some seasonal studies conducted on conifer species in temperate regions, showing differences in seasonal monoterpene emission patterns (Table 1). These reports often show increased emissions during warmer times of the year, particularly in late spring to mid-summer.

Though measurements of monoterpene emission rates were conducted for ambient temperatures, the normalized values (*β*-Factors) are given for a given standard temperature (30 °C) using the following algorithm developed by Guenther et al. [52].

E = Ms × exp [*β*(T − Ts)] (1)

where E is the monoterpene emission rate, T is the ambient/enclosure temperature, Ms is the emission rate at 30 °C, Ts is the standard temperature (303 K), and *β* is a parameter that accounts for the strength of the temperature dependence of monoterpene emissions for a given plant. The normalized values (*β*-Factors) reported in different studies are summarized in Table 1.

Monoterpene seasonal emission patterns seem to vary depending on the species, with some plants having high emissions in fall (*Pinus koraiensis*) or winter (*P. rigida*). We acknowledge the challenges in comparing reports due to the different practices in collection methods, analytical tools used, and unique environmental conditions at each collection site (as noted by two different studies conducted in Finland with *P. sylvestris)*. In the following paragraphs, we discuss some reports and their findings.

Monoterpene emissions for the Scots pine (*Pinus sylvestris* L.), a typical central European conifer, were measured during April, July, September, and October. The highest and lowest standard emission rates of the sum of total monoterpenes of 3739 ng g(dw)^−1^ h^−1^ and 240 ng g(dw)^−1^ h^−1^ were found in the months of April and July, respectively. The main contributor among all the individual sums of monoterpenes was 3-carene (42%), followed by *α*-pinene (30%), and *β*-pinene (15%), and contributions of other compounds were only 5% [116]. However, the emission rate of 1,8-cineole exhibited a different seasonality with the highest in April but the lowest in October. This suggests that individual monoterpene emissions may have a different seasonal dependence.

Three individual trees (Hinoki I, Hinoki II, and Hinoki III) of *Chamaecyparis obtusa* (Siebold and Zucc.) Endl. (the most dominant conifer tree species in Japan) exhibited significant changes in basal (standard) monoterpene emission rates with the season [126]. Their emission and composition (major compounds include sabinene, myrcene, and *p*-cymene) trends were almost similar. The highest basal emissions were observed during the winter, followed by autumn, spring, and summer in all three trees. The authors suggested that monoterpenes are stored in large pools in the leaves, which would increase the emission potential under lower temperatures in winter. The researchers also discovered that during the spring, the reproductive stage of the plant (*C. obtusa*) uses the majority of the photosynthetic products, while a smaller amount is used for monoterpene synthesis. In the summer season, the temperature is high and there may be dry conditions, so the photosynthetic rate is low and the monoterpene emissions from the pools are forced by evaporation, which makes monoterpene pools smaller in size, which could explain the low emissions. Therefore, the investigators concluded that monoterpene emission from *C. obtusa* depends both on monoterpene pools as well as reproductive stages. A similar trend was also observed in *Cryptomeria japonica* by Matsunaga et al. [119].

The emissions from two coniferous trees (*Pinus rigida* Mill.) and (*Pinus koraiensis* Siebold & Zucc.) show maximum emissions (30–50% of annual values) during spring and low emissions (2.98–3.2%). In spring, the correlation with environmental temperature was r^2^ = 0.786. While the emissions during summer and fall show almost similar values of 27.81–34.4% and 25.82–32.26%, respectively [125]. For the seasonal patterns of the emissions from *P. rigida* and *P. koraiensis,* the authors suggest the role of strong seasonality in temperature (spring: 20–22 °C, summer: 9–17 °C, fall: 11–12 °C, and winter: 4–13 °C) and their strong correlations with the emission rates. Mochizuki et al. [56] also reported the seasonal variation of standard monoterpene emissions of *Acer palmatum* Thunb., a mature tree in Japan with the maximum emission rates during the summer months (July–August).

The monoterpene emission rates measured from four chemotypes of *Cinnamomum camphora* L. during May (spring), July (summer), and November (autumn) show the highest values during the month of July [124]. Linalool was the dominant monoterpene from all four chemotypes during July, comprising 50–70% of all detected monoterpenes (such as eucalyptol, camphor, and endo-borneol). The authors noted that the seasonal variation could be due to changes in temperature and the expression of genes for monoterpene biosynthesis. The most emblematic forest species in central and northern Europe, the Norway spruce (*Picea abies* L.), showed a significant seasonal fluctuation in MT emission [127]. This study demonstrated a close relationship between solar radiation intensity (PAR) and camphene, limonene, and *α*- and *β*-pinene (predominant MTs). However, both PAR and temperature were found to stimulate delta-3-carene fluxes.

A field study on the native plant *Leptospermum scoparium* (mānuka) in New Zealand also revealed significant seasonal differences in monoterpene emission [27], with higher emission rates in the summer season. This study highlights the impact of biotic factors during different seasons, e.g., incidence of herbivore attack, the changing reproductive state of the plant (and changes in resource allocation), and the effect of neighboring plant species. While biotic factors are out of the scope of this review, in the future, we hope that developing technologies and trans-disciplinary research allow for a better insight of how complex interacting biotic and abiotic factors influence monoterpene emissions throughout the seasons and lifespan of the plant.

In general, there is still limited information available regarding the mechanisms behind the influence of environmental factors on terpene emissions. Studies show that temperature, vapor pressure of the terpenes, the humidity of the air surrounding the leaf, and the exposure area of essential oils are all involved in the passive release of constitutive terpenes, in a manner that is often independent from the stomatal opening, e.g., [128,129]. However, light does affect monoterpene production and subsequent release because it relies on photosynthetic products. Monoterpenes are also well-known antioxidants and are induced at a genetic level in response to heat, herbivory/damage, and radiation (oxidative stressors) through the action of secondary messengers such as reactive oxygen species or jasmonic acid, which trigger signaling cascades [130,131]. Interestingly, the presence of other antioxidants around the leaf surface, such as isoprene, also appears to have a regulatory role on monoterpene production that requires further elucidation [132]. Recently, numerous multi-substrate terpene synthases have been shown to exist based on recent improvements in the characterization of genes and enzymes responsible for substrate and end product biosynthesis [133]. An exciting recent study shows that enantiomers of the same compound (monoterpene) may behave differently in terms of production and release, and therefore mechanisms may vary depending on the enantiomeric distribution in a given plant species [134]. Last, but not least, plant phenology plays an important role in monoterpene production and release (due to trade-offs), along with factors influencing passive release and those causing increased production in response to stressors [135].

## 4. Analytical Options for Determining Emission Levels of Monoterpenes

Measuring the fluxes of low-volatility and highly reactive compounds like monoterpenes in different environments using conventional micrometeorological techniques is a challenging task. These specific BVOCs have short lifetimes in the atmosphere (ranging from seconds to minutes), resulting in low atmospheric concentrations and above-canopy fluxes. To measure these compounds at ambient levels, preconcentration from the surrounding air is typically necessary.

**Figure 2:**
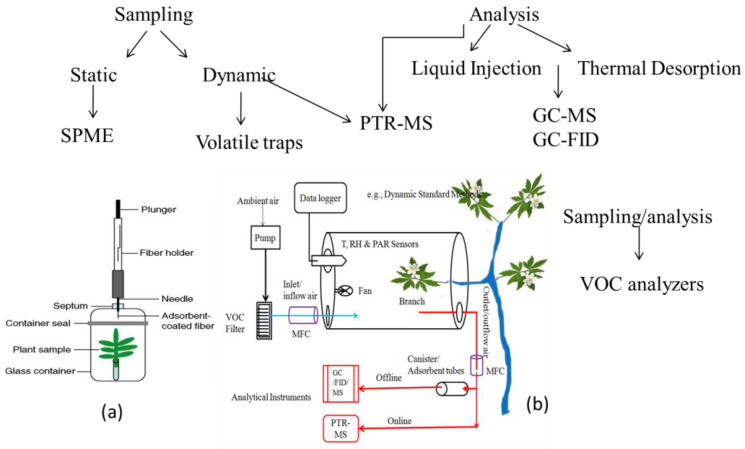
A schematic diagram of common analytical methods for determination of emission levels of monoterpenes from plants. (**a**) Static chamber method. (**b**) Dynamic enclosure chamber approach. (See text for abbreviations: Section 4).

Additionally, analytical losses can affect the measurement of reactive and low-volatility BVOCs. Due to these difficulties, accurately measuring the fluxes of reactive BVOCs at the canopy scale has seen limited success [136] and references therein. For instance, where ambient measurements are unfeasible, experiments involving enclosures at the leaf and branch levels offer viable alternatives for assessing fluxes of reactive and low-volatility compounds (Figure 2). Measuring branch-level emissions of monoterpenes presents even greater challenges. Nonetheless, despite the complexity, quantifying emission rates of these BVOCs from enclosures can serve as a basis for estimating overall canopy fluxes and their roles in atmospheric processes. This can be achieved by scaling emission rates observed in enclosure studies to the canopy level using precise site-specific biomass data and meteorological input parameters, e.g., [137]. In contrast to standardized procedures and commercial enclosure systems available for studying leaf-level photosynthesis and respiration, no such standardized methods exist for quantitatively measuring BVOC emissions. Most researchers construct their own apparatus and employ unique methods, making it challenging to compare reported emission rates across different studies.

Enclosures for vegetation experimentation can be categorized as static (no air flow) or dynamic (flow-through). In the static method, BVOC concentrations increase over time after enclosure installation. Emission rates are calculated by dividing the change in BVOCs concentration (emitted from enclosed foliage) by the duration of enclosure and the mass of leaves. Since air is not circulated, CO_2_ concentration tends to vary due to photosynthetic uptake, and greenhouse heating can lead to elevated temperatures. These conditions, with non-realistic CO_2_ concentrations, create artificial settings unsuitable for measuring naturally occurring emission rates. Short-term emission bursts caused by stress can also occur soon after enclosure setup, further complicating measurements. Static enclosures cannot be kept in place for extended periods, which introduces the risk of capturing artificially elevated emissions. Consequently, static methods are inadequate for estimating realistic long-term emission rates or diurnal variations. However, headspace sampling of static enclosures can be valuable for identifying BVOC emissions and developing analytical techniques, e.g., [138]. Solid-Phase Microextraction (SPME) is a sampling technique that involves the extraction of volatile compounds from the headspace of plant samples using a solid-phase fiber coated with an adsorbent. The compounds absorbed on the fiber are then desorbed and analyzed using techniques like gas chromatography (GC). SPME is a simple, solvent-free method, but it may suffer from limited sensitivity and selectivity for complex mixtures of monoterpenes.

Unlike static enclosures, dynamic enclosures allow for controlled environmental conditions and airflow [139]. More accurate emission rates can be derived from dynamic enclosures where air circulates around the vegetation. This maintains environmental parameters (temperature, CO_2_, PAR, and water vapor) relatively constant and closer to ambient levels, resulting in an enclosure environment that better represents natural conditions. The emission rate from a dynamic enclosure is calculated using empirical formulas [140]. In static chambers, temperatures rise without proper airflow, potentially impacting accurate BVOC estimations, including monoterpenes. Such uncertainties are minimized in dynamic chambers, where the absence of a purge flow is not mandatory. As such, dynamic chambers have become the preferred technique for measuring BVOCs from plant branches due to their convenience [141,142]. Despite the widespread adoption of dynamic enclosures, they have limitations. For instance, the materials used for chamber design, such as neoprene and low-density polyethylene polymers, are suspected to adsorb BVOCs, potentially leading to emission rate underestimations [140]. Enclosure experiments have been conducted on naturally growing vegetation as well as in greenhouses. Greenhouse experiments offer advantages, as controlling experimental conditions is generally easier indoors than outdoors. Operating analytical instruments indoors is more straightforward, facilitating the use of direct, online analysis techniques. However, due to space limitations, indoor emission studies are usually limited to smaller plants and the early growth stages of larger vegetation (such as saplings of larger trees). While greenhouse experiments can reveal correlations between emissions and environmental controls, it remains uncertain how these relationships translate to naturally growing vegetation. Consequently, when aiming to determine ambient flux estimates, conducting emission rate studies on naturally growing vegetation is preferable.

After sampling, various analytical techniques, including GC-flame ionization detection (FID), GC-mass spectrometry (MS), proton transfer reactive mass spectrometry (PTR-MS), and VOC analyzers, are employed for BVOC analysis. GC provides high sensitivity and selectivity, enabling the identification and quantification of multiple monoterpenes in a single analysis. However, sample preparation and complex chromatograms can be challenging, and some thermally labile compounds may degrade during the analysis. PTR-MS is a real-time, sensitive method that allows for the direct analysis of volatile compounds in the gas phase. It works based on the ionization of analytes by proton transfer reactions, followed by mass spectrometric analysis. PTR-MS offers high temporal resolution and sensitivity, making it ideal for studying fast-changing emission patterns. However, PTR-MS has limited compound identification capabilities, and the quantification can be challenging without proper standards. PTR-time of flight (ToF)-MS is an advanced version of PTR-MS, which couples proton transfer ionization with time-of-flight mass spectrometry. This method allows for high-resolution mass analysis and can identify and quantify a broad range of volatile compounds, including monoterpenes, with high sensitivity and accuracy. The main limitation is the high cost and technical complexity of the equipment.

Accurate determination of monoterpene emissions from plants is essential for understanding their ecological role and impacts on the environment. Each analytical method has its advantages and limitations, and the choice of technique depends on the research objectives, sample characteristics, and available resources. Combining multiple methods can provide complementary data and improve the overall understanding of monoterpene emissions. As technology continues to advance, new and more efficient methods for analyzing plant emissions will likely emerge, contributing to further insights into the complex interactions between plants and their environment.

## 5. Concluding Remarks

Monoterpenes are important compounds due to their ecological roles and contribution to atmospheric chemistry. Compounds such as *α*-pinene, *β*-pinene, limonene, *β*-myrcene, and *β*-ocimene appear to be widespread in terrestrial plants, but the bulk of knowledge is on temperate trees and grasses, so there is a need to investigate more tropical species and other plants such as ornamentals or invasives.

Numerous studies have explored monoterpene emissions from different terrestrial plant species, most notably for temperate tree species. However, comparing different reports is challenging due to different methods being employed for collection, analysis, and reporting units (e.g., use of single leaves vs. branches, headspace collection vs. solvent extraction, different analytical tools, reports based on dry weight vs. fresh weight, etc.). Moving forward, more standardized approaches or multi-species comparisons using the same methods would be useful for comparison purposes and to identify trends or patterns regarding their emission.

The studies reviewed in this paper clearly indicate that temperature and sunlight are critical factors influencing monoterpene emission, while the effects of other abiotic factors (ozone exposure, soil moisture, etc.) are less clear. Differences between monoterpene storing and non-storing species remain to be further explored.

Monoterpene emissions have seasonal patterns showing increased emissions during warmer times of the year, particularly in late spring to mid-summer. However, the complexity of interacting biotic and abiotic factors involved in seasonal emissions is far from fully understood. It is likely that new technologies will be of assistance in advancing the collection and analyses of complex datasets.

## Figures and Tables

**Table 1:** Normalized emissions (*β*-Factor) for monoterpenes released from different conifer species during different seasons, with temperature ranges (if provided). The *β*-Factor is a normalized value of emission given a standard temperature of 30 °C, following Guenther et al. [52].

Plant Species	*β*-Factor (K^−1^)	Season	Temperature Range (°C)	Ref
*Pinus densiflora*	0.18	Spring	Not given	[117]
	0.14	Summer		
	0.06	Fall		
	0.05	Winter		
*Pinus rigida*	0.07	Spring	22–42	[125]
	0.04	Summer	23–40	
	0.03	Fall	10–22	
	0.08	Winter	11–15	
*Larix leptolepis*	0.14	Spring	Not given	[49]
	0.14	Summer		
	0.07	Fall		
	n/a	Winter		
*Pinus koraiensis*	0.26	Spring	23–45	[125]
	0.09	Summer	26–35	
	0.18	Fall	18–29	
	0.08	Winter	5–18	
*Pinus sylvestris* ^1^	0.13	Early Spring	Not given	[120]
	0.08	Late spring		
	0.15	Summer		
*Pinus sylvestris* ^2^	0.10	Spring	Not given	[120]
	0.18	Early Summer		
	0.08	Late Summer		
	0.11	Autumn		
*Chamaecyparis obtusa*	0.08–0.35	Winter	Not given	[126]
	0.07–0.12	Spring		
	0.13–0.15	Summer		
	0.024–0.16	Autumn		

^1^ Finnish Lapland in Sodankyla. ^2^ Southern Finland in Hyytial.

## Data Availability

Not applicable.
